# Effects of Cast-Iron Surface Texturing on the Anti-Scuffing Performance under Starved Lubrication

**DOI:** 10.3390/ma12101586

**Published:** 2019-05-15

**Authors:** Wenhua Li, Baihong Yu, Bin Ye, Yan Shen, Ruoxuan Huang, Fengming Du

**Affiliations:** 1 Marine Engineering College, Dalian Maritime University, Dalian 116026, China; lwh992@dlmu.edu.cn (W.L.); 18041103373@163.com (B.H.Y.); nomele@foxmail.com (B.Y.); dfm@dlmu.edu.cn (F.D.); 2 Key Laboratory of Ship-Machinery Maintenance & Manufacture, Dalian Maritime University, Dalian 116026, China

**Keywords:** cast iron, cylinder liner, micro-dimple, friction coefficient, anti-scuffing, starvation

## Abstract

Advances in heavy-duty diesel engine designs place higher demands on the friction and wear performance of the piston ring and cylinder liner (PRCL) interface. The potential of using micro-textures machined on the whole stroke of a cast-iron cylinder liner was investigated in this work. A set of running-in and starved lubrication experiments was performed using a custom reciprocating test rig that imparts a combination of combustion-level pressures and the resulting impacts. Based on a comparison of micro-dimple parameters, the friction coefficient for the running-in period at the shocking dead center was the smallest at a designed combination of 1000-μm diameter, 22% area fraction, and arrangement with half-radius intersecting distance of two adjacent micro-dimple columns. The non-scuffing time under starvation was the longest at a designed combination of the following parameters: 800 μm diameter, 22% area fraction, and quarter-radius intersecting distance arrangement. From finite element analysis, it was found that stress concentrates at the micro-dimple periphery and at the connections between adjacent micro-dimples. However, surface topography examination showed that scuffing initiates in the non-dimpled regions between the micro-dimpled columns rather than at their edges. Finally, under reciprocating motion, micro-dimples can collect wear debris to inhibit further propagation of scuffing in the micro-dimpled region.

## 1. Introduction

As the explosive pressure in heavy-duty diesel engines increased to more than 20 MPa, the lubrication state existing in the interface of the piston ring and cylinder liner (PRCL) accordingly became even worse than ever before. The possibility of direct contact among the micro-asperities will increase significantly, resulting in increased mechanical power loss and wear failure, as well as seizure problems in the power cylinder components [1,2].

Cylinder scuffing is generally considered to be an abrupt rise in the friction coefficient, accompanied by apparent damage on the cylinder liner surface [3,4,5,6]. Many efforts were made to investigate the scuffing phenomenon to prevent such failure [7,8,9,10,11,12]. Coating on the piston ring proved to be an effective method, which may provide advantages such as friction reduction and improved wear and scuffing resistance. Shen et al. compared heavy-duty scuffing behavior between chromium-based ceramic composite and nickel–chromium–molybdenum-coated ring sliding against a cast-iron liner [13]. Davis et al. showed that 20 wt.% Cr_2_AlC-blended Ni–Mo coating on the piston ring presented low wear rate and friction coefficient in comparison to other coatings [14]. Wan et al. reported that the conventional ceramic coating embedded with hard amorphous graphite-like carbon was an economical candidate with respect to good scuffing resistance [15]. Lin et al. prepared TiSiCN nanocomposite coatings, which showed an obvious reduction in the ring weight loss for the coated top and second rings [16]. 

To further improve the tribological performance of the PRCL, surface modification was recently carried out on the cylinder surface as well. One of the most prevalent strategies is micro-texturing [17,18]. The micro-textured morphology can be fabricated as the non-cross and non-connected micro-dimple arrays on the cylinder liner surface [19]. Etsion et al. proposed a model to study the potential use of micro-surface structure in the formation of micro pores, and the pores can maintain hydrodynamic effects and substantially reduce the friction losses in reciprocating automotive components [20,21]. Nakano et al. indicated that the dimpled pattern had a beneficial effect of decreasing the friction under lubricated conditions [22]. Ramesh et al. reported that the friction force of the textured surfaces can be reduced by 80% compared to the untextured surfaces during hydrodynamic lubricated sliding [23]. These studies showed that the micro-texture can enhance friction reduction effect under the hydrodynamic lubrication condition. In recent years, the micro-texture morphology was introduced to improve the friction and wear performance of the PRCL at the top dead center where the direct contact of the asperities usually occurs. Generally, it is believed that micro-textures have the following advantages: (a) Storage of the wear debris to avoid the abrasive wear on the contact surface [24,25]; (b) Retention of the lubricating oil to enhance the hydrodynamic lubrication effect [26,27]; and (c) Reduction of the direct contact area of the mating pair [28]. However, the unsuitable micro-textures may play a negative role on lubricating oil supplement to the contact area of the micro-asperities, which will then result in a significant friction coefficient increase [29]. It is necessary to optimize the design of micro-texture parameters to meet the harsh operating conditions of heavy-duty diesel engines. In addition, scuffing behavior of the micro-dimpled surface under starvation should not be neglected either.

Most of the investigations adopted the laser texturing technology to prepare the micro-textured surfaces, but a high temperature is prone to causing material accumulation during the laser ablation [30,31]. High temperature generated by the laser etching would also change the material properties of the mating pair. The worn, hard, abrasive particles would cause surface damage and deteriorate the friction state. In this work, the reciprocating electrolyte jet with prefabricated mask (REJP) machining technology was used to machine the micro-dimpled textures instead of laser texturing [32]. The prefabricated mask with a purposely designed micro-texture pattern was pasted on the cylinder liner inner surface to determine the area to be electrolytically etched. The electrolyte from the cathode jet was spread out to the prefabricated mask surface via the reciprocating motion of the cathode. The uncovered cylinder liner area by the prefabricated mask would generate the electrolytic action with satisfactory control of the micro-machining process. Compared with the non-dimpled surface, the micro-dimpled textures on the cylinder surface have a good friction reduction effect. In this paper, the anti-scuffing behavior of the micro-dimpled cylinder liner was further studied.

Cast iron is a prevalent material in fabricating diesel engine cylinder liners due to its low cost and good wear resistance property. In order to improve the reliability of the cast-iron cylinder liner under highly strengthened conditions, the appropriate micro-textured parameters should be obtained on the cylinder liner surface using starvation condition experiments. The PRCL interface was imparted a combination of combustion-level pressures and the resulting impacts to simulate explosion pressure effects. A set of running-in and starved lubrication experiments were designed to evaluate the anti-scuffing performance of cast-iron cylinder liners with different micro-dimpled textures. Based on a comparison of the friction coefficient and the non-scuffing time, the micro-dimple parameters could be optimized. These results may provide design guidance to a certain extent to improve the anti-scuffing performance for the PRCL.

## 2. Experimental Details

### 2.1. Test Rig Description

**Figure 1:**
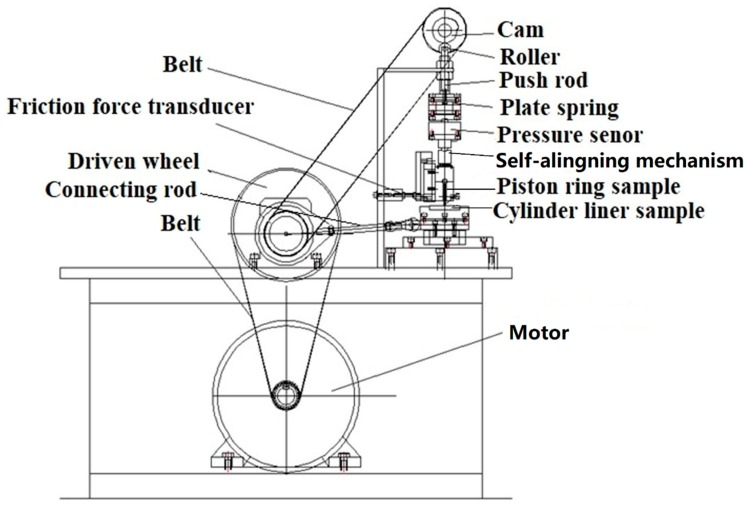
Piston ring reciprocating cylinder liner (PRCL) test rig for the explosive pressure impact simulation.

The newly designed piston ring reciprocating liner test rig was developed based on the original test rig [13], as illustrated in Figure 1. The driving force generated from the motor rotates the crank through the reduction gear, and then the crank-connecting rod mechanism transforms rotary motion into reciprocating motion of the cylinder liner sample. Meanwhile, the driven wheel drives the cam to rotate through the belt. A cam-roller loading mechanism through the plate spring is applied to exert shock loading on the dead center interface between moving parts (cylinder liner sample) and fixed parts (piston ring sample). This mechanism can simulate a combination of combustion-level pressures and the resulting impacts to the PRCL interface. The self-aligning mechanism between pressure sensor and piston ring sample could evenly transmit the nominal pressure across the ring/liner in the circumferential direction. When the piston ring sample and cylinder liner sample had the relative sliding motion, a piezoelectric force transducer mounted behind the piston ring fixture was subjected to a tension–compression action, allowing the friction force to be recorded online in the experiment. The test rig can accommodate a wide range of nominal pressures (5 MPa–200 MPa), displacement frequencies (1 Hz–40 Hz), and temperatures (25 °C–300 °C) at the PRCL interface. Lubricating oil was applied to the interface through a peristaltic pump at the dosage of 0.1 mL/min. The oil was evenly distributed along the entire 30-mm stroke.

**Figure 2:**
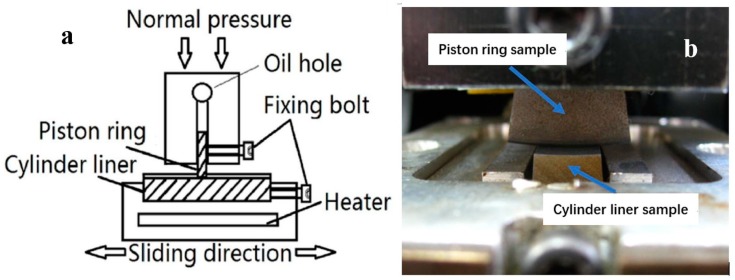
Detailed layout of piston ring and cylinder liner (**a**) and the PRCL contact along the circumferential direction (**b**).

In order to ensure good contact between the piston ring and the cylinder liner, the loading mechanism through the plate spring was applied to exert the steady normal pressure on the interface between moving parts (cylinder liner) and fixed parts (piston ring), as shown in Figure 2a. The self-aligning mechanism can evenly transmit the nominal pressure across the ring/liner in the circumferential direction. This can adequately guarantee the conformity of the samples across the ring/liner in the circumferential direction, as shown in Figure 2b. The outer circumference of the piston ring sample conforms well with the inner circumference of cylinder liner sample.

In the whole stroke of a real engine, the PRCL will experience fluid lubrication, mixed lubrication, and boundary lubrication. When the lubrication state deteriorates, wear damage will occur due to the mutual contact of micro-asperities, particularly at the top dead center. The focus in this paper is the friction and wear problem of the PRCL, whereby we shorten the stroke length of test rig to simulate the wear process in the top dead center area. Although load and temperature of the PRCL interface vary instantaneously with the piston position in a real engine, the most severe wear area of the PRCL is still in the vicinity area of top dead center. Constant setting of test conditions in the test rig is helpful to compare the friction and wear performance of different surface micro-textures. In addition, in order to accelerate the wear process and reduce the test period, a certain explosion pressure condition was applied to simulate the load in the PRCL contact area of the test rig. 

### 2.2. Experimental Materials

The piston ring had an inner diameter of 70 mm, an outer diameter of 110 mm, and a thickness of 3 mm. It was cut into 20 equal portions along the circumference as the piston ring sample. The piston ring was coated with the chromium-based ceramic composite coating (CKS) with a roughness Ra (arithmetical mean deviation) of 0.24 μm. The actual plateau honed boron–phosphorus alloy cast iron was selected as the cylinder liner sample. Its bearing ratio parameters are listed in Table 1. The roughness Ra was 0.72 μm. The inner diameter was identical to the outer diameter of the ring sample, resulting in perfect mating between the friction pair. The cylinder liner was cut into 40 equal portions along the circumference, and then the micro-dimpled textures were electrolytically machined on the inner surface of the cylinder liner samples using the REJP technology [32]. Some tested material parameters of cast-iron liner and CKS ring are listed in Table 2. The lubricant was 10W-30 CD diesel oil, which was commercially available at the Great Wall Lubricant Corporation of China (Beijing, China). The viscosity index of this oil was 138. The viscosity was 114.9 mm^2^/s at 40 °C and 16.35 mm^2^/s at 100 °C, respectively. The extreme pressure additives were mainly zinc dialkyl dithiophosphates (ZDDP).

### 2.3. Micro-Dimple Parameters

**Figure 3:**
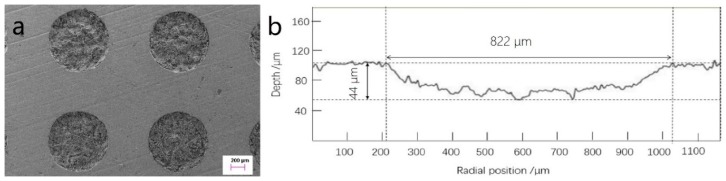
Surface topography of micro-dimpled cylinder liner (**a**) and two-dimensional (2D) profile of single micro-dimple (**b**).

**Figure 4:**
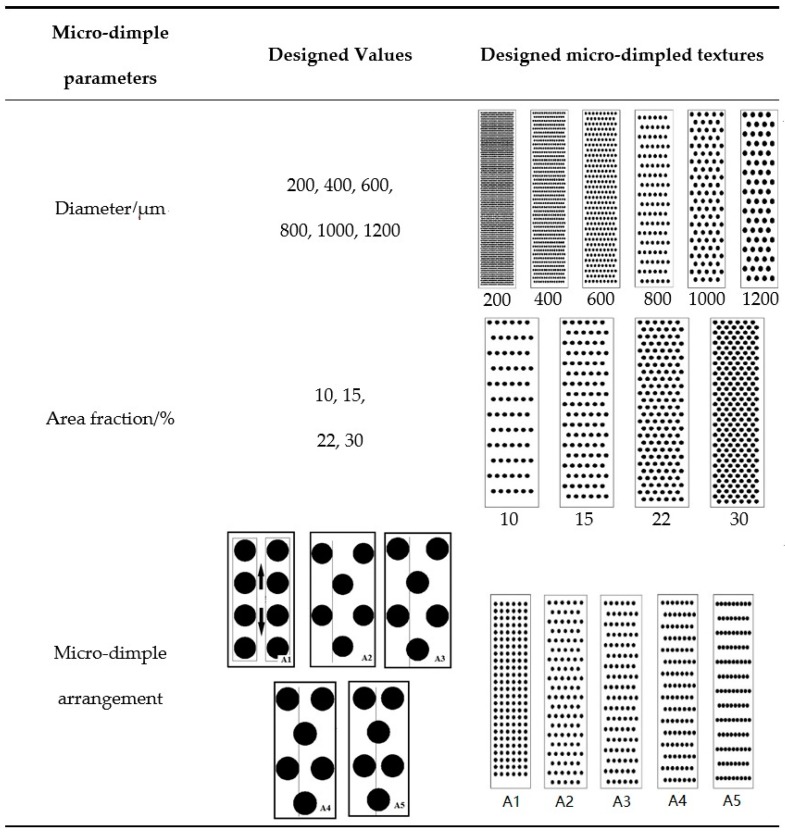
The designed parameters of micro-dimpled textures on the cylinder liner.

Figure 3 shows the surface topography of the micro-dimpled cylinder liner and a two-dimensional (2D) profile of a single micro-dimple. It presents a regular shape with the parameters of ~822 μm in diameter and ~44 μm in depth. Compared with the laser texturing technology, REJP machining technology without high-temperature evaporation can retain the graphite sheets in micro-dimpled textures. These graphite sheets are helpful to form the solid lubrication at the contact area [32]. Based on previous literature [33,34,35], the designed micro-dimple parameters on the cast-iron cylinder liner which probably affect the scuffing resistance behavior, including micro-dimple diameter, area fraction, and micro-dimple arrangement, are shown in Figure 4. The designed diameters of the circular micro-dimple varied from 200 μm to 1200 μm. The area fractions defined as the ratio of the micro-dimple area to the cylinder liner sample area were 10%, 15%, 22%, and 30%. The arrangements of micro-dimpled textures were distinguished by the relative position of the two adjacent micro-dimple columns as follows: A1 means that the two adjacent columns of the micro-dimple along the sliding direction were separated by a certain distance of ~550 μm; A2 means that the position of the two adjacent columns of the micro-dimple along the sliding direction was tangential; A3, A4, and A5 represent that the two adjacent columns of the micro-dimple along the sliding direction intersected, and the intersecting distances were one-quarter, one-half, and three-quarters of the radius, respectively.

### 2.4. Experimental Procedure

The running-in and starved lubrication experiment was divided into four stages: the running-in stage with the light load (RLL), the running-in stage with the heavy load (RHL), the heating stage (HS), and the oil starvation stage (OS). The running-in stage was supplied with adequate lubricating oil at the dosage of 0.1 mL/min. The oil was evenly distributed along the entire 30-mm stroke. The maximum nominal pressure of 50 MPa corresponds to a maximum normal force of 1280 N at the shocking dead center. The RLL was to eliminate large burrs. The RHL was used to form a relatively stable contact state on the PRCL interface; then, the HS was applied to accelerate the simulation process of the cylinder scuffing and improve the test efficiency. The OS was to stop the lubricating oil (LO) supply and form the starved lubrication condition at the PRCL interface. The experimental conditions are shown in Table 3. For each test, the frequency of reciprocating motions of the cylinder liner was set at 6.67 Hz.

**Figure 5:**
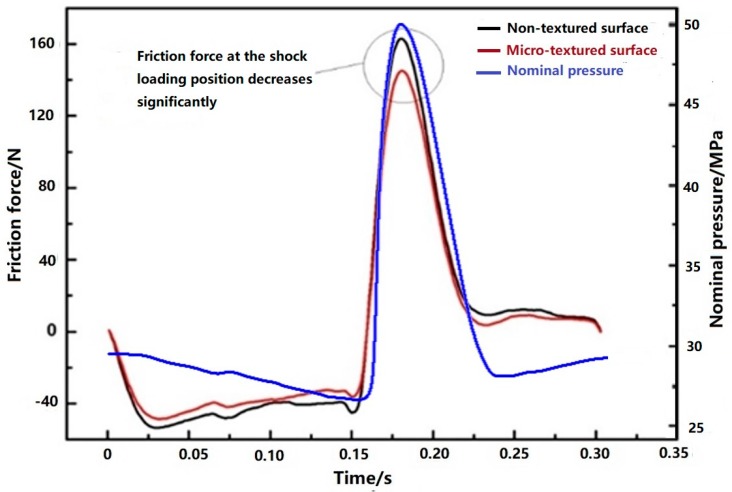
Friction force comparison and nominal pressure variation of a typical reciprocating stroke in the stable wear period.

Figure 5 shows the friction force comparison and nominal pressure variation of a typical reciprocating stroke in the stable wear period at the measurement frequency of 1000 Hz. A cam-roller loading mechanism generated the shock loading at one of the dead center positions. This action caused the friction force to be asymmetrical when moving back and forth. The measured friction force was filtered by the empirical mode decomposition method [36,37]. The existence of micro-texture can significantly reduce the friction force between cylinder liner and piston ring during the whole stroke, especially at the shock loading position. 

**Figure 6:**
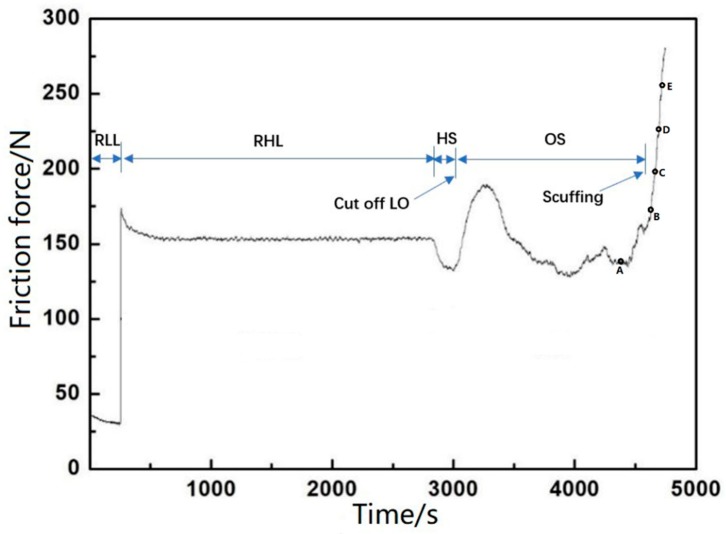
Typical maximum friction force variation in the running-in and starved lubrication experiment.

**Figure 7:**
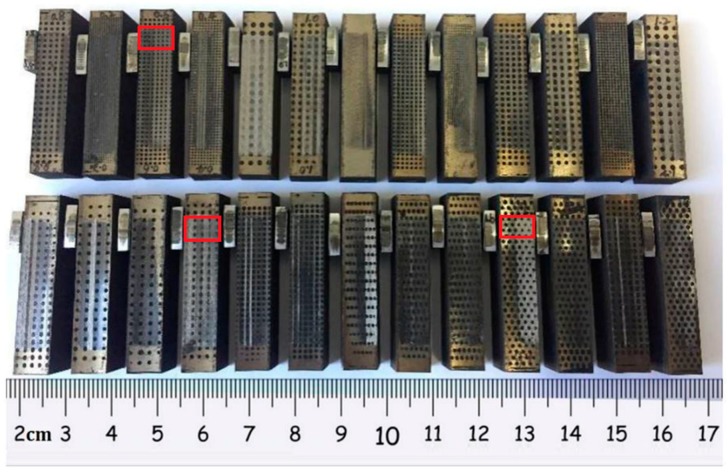
The typical tested PRCL samples.

Figure 6 shows the typical maximum friction force variation at the dead center in the running-in and starved lubrication experiment. As the applied normal force between the PRCL is known, the friction coefficient at the shocking dead center was calculated from the ratio of the largest friction force to maximum normal force. Since the shocking dead center is the main concern, we only present the friction coefficient of the micro-textured surface at the shock loading position. One can see the comparison of the friction coefficient variation at the shocking dead center. It was taken from the late RHL stage before the HS stage. The non-scuffing time was defined as the time interval between the lubrication oil supply stopping and the moment of the scuffing emerging. Phase space trajectories extracted from the transient features of the friction force were used to judge the occurrence of scuffing [38]. Compared with the friction force of the previous whole stroke, the friction force in the whole stroke presented a sudden drop before scuffing. Shortly after this moment, the scuffing was found to start. For each micro-dimpled texture parameter, at least three repeat tests were carried out. The average and standard deviation were calculated from the three results. Figure 7 presents the typical tested PRCL samples. The position of cylinder liner in the red frame corresponds to the shock loading position at which the greatest friction force occurs in the reciprocating motion. At the end of the RHL stage, the wear amount of the frictional pair was too small to be measured; thus, the friction coefficient at the shocking dead center and non-scuffing time were used to evaluate the effect of the micro-dimpled textures on the scuffing performance.

In addition, the arithmetical mean deviation (Ra) of surface roughness measurements was performed using an OLYMPUS LEXT OLS3100 (Olympus Corporation, Tokyo, Japan). The measurement area was 256 × 256 μm^2^, which had a total of 1024 × 1024 sampling points with a maximum height resolution of 0.01 um. The worn and unworn surfaces were examined using a SUPRA 55 SAPPHIRE scanning electron microscope (SEM, Carl Zeiss NTS GmbH, Oberkochen, Germany) and energy-dispersive X-ray spectroscopy (EDS, Carl Zeiss NTS GmbH, Oberkochen, Germany).

## 3. Results and Discussion

### 3.1. Effect of the Micro-Dimple Diameter on the Friction Coefficient and Non-Scuffing Time

**Figure 8:**
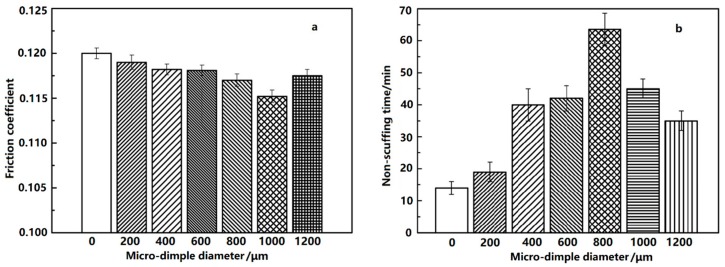
Friction coefficient at the shocking dead center (**a**) and non-scuffing time (**b**) with different micro-dimple diameters.

Compared with the non-dimpled cylinder liner, the friction coefficient at the shocking dead center and the non-scuffing time with the various designed micro-dimple diameters are shown in Figure 8. The other geometric parameters were kept constant (22% area fraction and A3 arrangement).

As can be seen from Figure 8a, the friction coefficient of the non-dimpled cylinder liner was greater than that of the micro-dimpled cylinder liner, implying that the existence of the micro-dimple on the cylinder surface is beneficial for reducing the friction loss of the PRCL. For the micro-dimpled textures with different diameters, the friction coefficient firstly decreased and then increased with the designed micro-dimple diameter increasing from about 200 μm to 1200 μm. When the designed micro-dimple diameter was 1000 μm, the friction coefficient was the smallest with a value about 3.19% lower than that of the non-dimpled cylinder liner.

As can be seen from Figure 8b, the non-scuffing time of non-dimpled cylinder liner showed shorter non-scuffing duration than that of the micro-dimpled cylinder liner, indicating that the existence of the micro-dimple arrays on the cylinder surface is beneficial for improving the scuffing resistance of the PRCL as well. For the micro-dimpled textures with different diameters, the variation of non-scuffing time firstly increased and then decreased with the designed micro-dimple diameter increasing from 200 μm to 1200 μm. Unlike the friction coefficient, the longest non-scuffing time was found at the designed micro-dimple diameter of 800 μm, and was about 4.5 times longer than that of the non-dimpled cylinder liner.

### 3.2. Effect of the Area Fraction on the Friction Coefficient and Non-Scuffing Time

**Figure 9:**
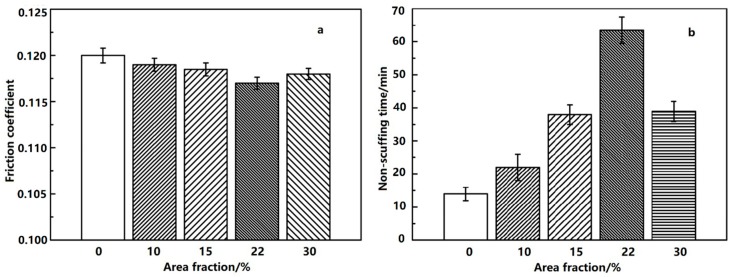
Friction coefficient at the shocking dead center (**a**) and non-scuffing time (**b**) with different area fractions.

Compared with the non-dimpled cylinder liner, the friction coefficient at the shocking dead center and the non-scuffing time with the various area fractions are shown in Figure 9. The other geometric parameters were kept constant (800 μm micro-dimple diameter and A3 arrangement).

The friction coefficient and non-scuffing time of the non-dimpled cylinder liner were not better than those of the micro-dimpled cylinder liner. The friction coefficient firstly decreased and then increased with the designed area fraction increasing from 10% to 30%. When the designed area fraction was 22%, the friction coefficient was the smallest. For the micro-dimpled textures with different area fractions, the variation of non-scuffing time presented the same trend as the micro-dimple diameters. However, the longest non-scuffing time was also observed at the designed area fraction of 22%.

### 3.3. Effect of the Micro-Dimple Arrangement on the Friction Coefficient and Non-Scuffing Time

**Figure 10:**
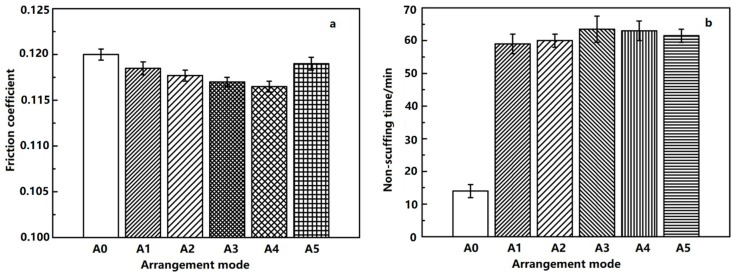
Friction coefficient at the shocking dead center (**a**) and non-scuffing time (**b**) with different micro-dimple arrangements.

Compared with the non-dimpled cylinder liner, the friction coefficient at the shocking dead center and the non-scuffing time with the different dimple arrangements at the designed micro-dimple diameter of 800 μm and area fraction of 22% are shown in Figure 10.

The friction coefficient and the non-scuffing time of non-dimpled cylinder liner (A0) was also not better than that of the micro-dimpled cylinder liner. The friction coefficient firstly decreased and then increased with the dimple arrangement varying from A1 to A5. When the dimple arrangement was A4, the friction coefficient was the smallest. For the micro-dimpled textures with different arrangements, the non-scuffing time of intersect arrangement (A3, A4, A5) was better than the separated (A1) and tangential arrangements (A2). The variation of non-scuffing time between A3 and A4 presented a little difference, but A3 was slightly longer than A4.

### 3.4. Contact Analysis of Micro-Dimpled Cylinder Liner with Finite Element Method (FEM)

A typical micro-dimpled surface provides scuffing analysis of the micro-dimpled cylinder liner at the designed combination of geometric parameters, i.e., micro-dimple diameter of 800 μm, area fraction of 22%, and A3 arrangement. FEM analysis was employed to acquire the contact stress distribution on the micro-dimpled cylinder liner without considering the influence of fluid lubrication.

From the friction coefficient comparisons at the shocking dead center, all values at the dead center were more than 0.1 at a load of 50 MPa. This value demonstrates that solid contact emerges on the micro-dimpled surface under the boundary lubrication state. In order to understand the effect of micro-dimpled surface during contact, the finite element software ANSYS was used to analyze the contact conditions.

The contact problem was simplified as a micro-dimpled cylindrical liner acting against a barrel-shaped ring with solid contact [39,40]. Surfaces of the liner and the ring were frictionless. The cast-iron liner worked as the cylindrical shape and restricted the degree of freedom at the bottom. The CKS ring with the symmetrical barrel shape had an axial thickness of 3 mm and a barrel surface height of 10 mm, just like the tested piston ring. The ring exerted a normal force. The contact mode was set as flexible–flexible contact. The inner surface of the cylinder liner and the outer surface of the piston ring were set as the contact surface and target surface, respectively. The element types were set as Conta174 and Targe170, respectively. Poisson’s ratio and Young’s modulus of the cast-iron liner and CKS ring are presented in Table 2.

Detailed parameters and conditions were as follows:

Area fraction r = 22%;
Micro-dimple diameter D = 800 μm;
Contact pressure P = 50 MPa;
Friction coefficient f = 0.10.

**Figure 11:**
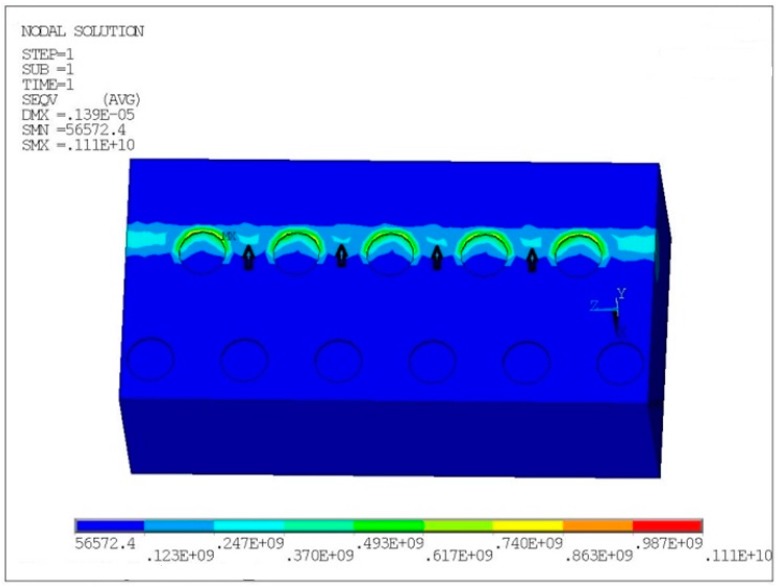
Mises stress distribution on the contact surface of micro-dimpled cylinder liner (space unit: μm; stress unit: Pa).

Figure 11 presents the contact stress distribution of the micro-dimpled cylinder liner in units of Pa. It was found that the existence of the micro-dimpled textures led to two types of contact stress concentration regions. One of the stress concentration regions was at the edge of the micro-dimple, while the other region was located at the connections between adjacent micro-dimples (black arrow). Compared with other regions, the surface damage would most likely initiate at the stress concentration regions during sliding.

### 3.5. Analysis of Anti-Scuffing Performance Variation at Different Micro-Dimpled Parameters

Based on the experimental results, the micro-dimple parameters of diameter and area fraction had the optimum combination in terms of anti-scuffing performance. The increased micro-dimple diameter and higher area fraction would reduce the contact area of the PRCL interface and contribute to lessening the direct contacts of asperities. Meanwhile, more lubricating oil storage in the dimple would give an additional supply source to the contact interface, where this action can delay the consumption of boundary lubrication film on the PRCL contact region. The comprehensive effect of these factors can improve the anti-scuffing and frictional behavior. However, the FEM analysis demonstrated that the stress concentration existed around the region of micro-dimple edge. This edge effect becomes obvious with the micro-dimple parameters exceeding the maximum limit value. An excessive stress concentration will cause the sliding surface to be more vulnerable to damage under starvation. There is a balance between the lubrication enhancement and stress concentration brought about by the micro-dimpled textures on the PRCL interface.

In addition to the micro-dimple diameter and area fraction, the micro-dimple arrangement mode also had an obvious effect on the anti-scuffing behavior. In the case of delaying the damage propagation on the micro-dimpled surface under starvation, the micro-dimple arrangement modes of A3 and A4 were much better than those of A1 and A2. The A1 and A2 arrangements of the micro-dimples could get the additional lubricating oil only from the adjacent micro-dimple along the circumference direction, but the A3 and A4 arrangements could add another source of oil supplement from the staggered micro-dimple along the sliding direction. Excessive misplacement, such as the A5 arrangement, may make the edge effect become obvious and weaken the effect of lubricant feeding. Therefore, proper staggered micro-dimples can effectively prevent the scuffing emerging prematurely at the connections between adjacent micro-dimples.

### 3.6. Scuffing Analysis of Micro-Dimpled Cylinder Liner

**Figure 12:**
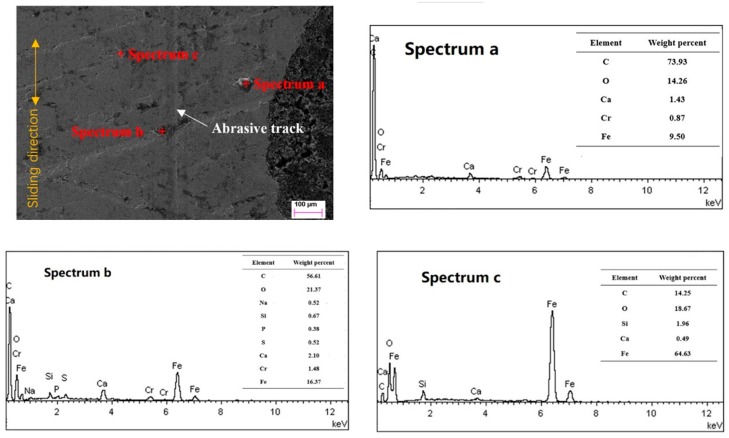
Topography and element composition on the left part of the micro-dimpled surface before scuffing.

In order to better understand scuffing process of micro-dimpled cylinder liner under the shock loading action, all measured locations on the cylinder liner were at the shock loading position of dead center. Figure 12 presents the left part surface of the non-scuffed micro-dimple at point A in Figure 6 before scuffing. The honing textures experienced a plastic flow under local high stresses and frictional heat. The shallow honing textures were slid flat, while only the deeper honing textures still existed. There was an abrasive track emerging adjacent to the micro-dimple. If micro-dimples can be staggered in the scratch zone, an abrasive track may be difficult to scratch across the entire observed micro-dimpled area. The experimental results prove that the staggered arrangements (A3, A4, A5) can delay the premature occurrence of the cylinder scuffing compared with the in-line arrangements (A1, A2). The EDS spectra a and b show that the element Cr from the CKS piston ring existed in the detected areas. It indicates that microscopic adhesions at the PRCL interface occurred. Based on the comparison of elements, spectrum a contained much more C element than spectrum b and spectrum c. This may have been caused by the graphite sheet being transferred from the micro-dimple to the vicinity region. S, P, and Ca from lubricating oil additives were observed in spectrum b. It indicates that a protective film was formed between the lubricant additives and micro-asperities by the tribo-chemical action [41,42].

**Figure 13:**
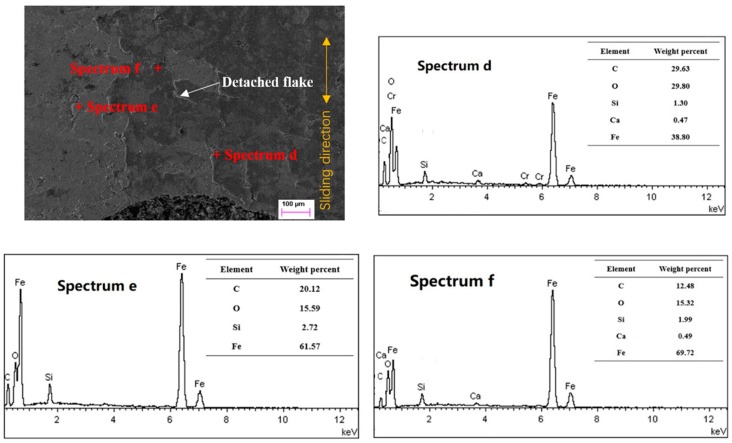
Topography and element composition on the upper part of the micro-dimpled surface before scuffing.

Figure 13 presents the upper part surface of the non-scuffed micro-dimple at point A in Figure 6 before scuffing. It can be seen that the honing surface of the cylinder liner was plastically deformed, and almost detached flakes existed in some areas. All the detected positions d, e, and f did not contain the Cr element from the piston ring. Based on the comparison of elements, spectrum d showed much more C element than spectrum e and spectrum f. It also indicates that the closer it is to the micro-dimple, the more C element exists. The graphite sheet from the micro-dimple may supply the graphite solid lubricant to the vicinity region under the reciprocated sliding motion.

**Figure 14:**
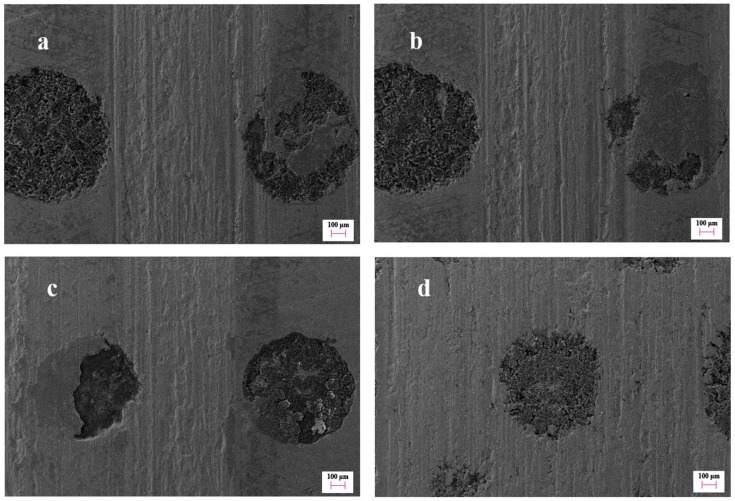
The topography variation of micro-dimpled cylinder liner at point B (**a**), point C (**b**), point D (**c**), and point E (**d**) in Figure 6 during the scuffing.

Figure 14 shows the topography variation of the scuffed micro-dimple at points B, C, D, and E in Figure 6 during scuffing. Serious damage originated in the non-dimpled region between the adjacent micro-dimples, as illustrated in Figure 14a. There was a marked groove along the sliding direction between the adjacent micro-dimples, but the micro-dimpled region presented a slight damage state. One of the micro-dimples was filled with some agglomerated materials. It indicates that the matrix material of the cylinder liner was rolled into the micro-dimple after plastic deformation. As the adhesive wear became serious, the abrasive particles gradually filled into the whole micro-dimple, as illustrated in Figure 14b.

Although the micro-dimple can collect the wear debris, the serious damage region gradually propagates to the micro-dimpled region. The scratch along the sliding direction began to appear on the micro-dimpled region, but the damage degree of the micro-dimpled region was lighter than that of the non-dimpled region, as illustrated in Figure 14c. As the scratches in the micro-dimpled region further expanded, the scratches were exacerbated to the entire surface, producing more wear debris in the micro-dimple, as shown in Figure 14d.

Compared with laser texturing [18,27,43], the electrochemical machining micro-dimples on the cast-iron cylinder liner surface supplied not only the lubricating oil, but also the graphite sheet to the surrounding area. These graphite sheets overflowing from the micro-dimple may form the solid lubrication state at PRCL interface. The closer proximity to the micro-dimple makes it much easier to replenish the lubricant consumption, which is helpful to prevent the scuffing emerging prematurely under starvation. Although the simulation results of finite element analysis indicated that the maximum stress was at the micro-dimple edge, the scuffing initiated at the connections between adjacent micro-dimples, and then gradually expanded to the micro-dimpled region. This may have been due to the presence of the lubricating medium around the micro-dimple and the wear debris storage effect of the micro-dimpled textures. After the scuffing emerging, the micro-dimple can collect the wear debris agglomeration to occupy the micro-dimple space and reduce the abrasive wear. 

## 4. Conclusions

The heavy-duty scuffing behavior of the micro-dimpled cylinder liner was investigated using the piston ring reciprocating liner test rig. The PRCL interface was starved of lubrication after the running-in period under the simulated combination of combustion-level pressures and the resulting impacts. The micro-dimpled parameters of diameter, area fraction, and arrangement mode presented a close effect on the scuffing resistance. The main conclusions are summarized below.

(1) Based on a comparison of micro-dimple parameters, the friction coefficient of running-in period at the shocking dead center was the smallest at the following designed geometric parameter combination: micro-dimple diameter of 1000 μm, area fraction of 22%, arrangement with half-radius intersecting distance of two adjacent micro-dimple columns), and the non-scuffing time under starvation was the longest at the following designed geometric parameter combination: micro-dimple diameter of 800 μm, area fraction of 22%, and quarter-radius intersecting distance arrangement;
(2) Under starvation, the scuffing initiated in the non-dimpled regions between the micro-dimpled columns rather than at their edges;
(3) Graphite sheets in the micro-dimples of the cast-iron cylinder liner may be released to the surrounding region under the reciprocated motion, suppressing the occurrence of severe scuffing;
(4) After the scuffing emerging, the micro-dimpled textures can collect the wear debris agglomeration to inhibit further propagation of scuffing in the micro-dimpled region.

## Figures and Tables

**Table 1:** Bearing ratio parameters of plateau honed cast-iron liner.

Bearing Ratio Parameters	Values
Core roughness/μm	1.26
Reduced peak height/μm	0.52
Reduced valley depth/μm	0.92
Upper material ratio	10.65
Lower material ratio	88.91

**Table 2:** Some material parameters of cast-iron liner and chromium-based ceramic composite coating (CKS) ring.

Material Parameters	Cast-Iron Liner	CKS Ring
Hardness (HV0.1)	238	705
Young’s modulus (GPa)	120	250
Poisson’s ratio	0.25	0.12

**Table 3:** Experimental conditions of the running-in and starved lubrication experiment. RLL—running-in stage with light load; RHL—running-in stage with heavy load; HS—heating stage; OS—oil starvation stage.

Experimental Stage	Maximum Nominal Pressure (MPa)	Temperature (°C)	Time (min)
RLL	10	150	~10
RHL	50	150	~85
HS	50	150 to 220	~5
OS	50	220	To scuffing
